# Thrombosis of A Prosthetic Mitral Valve After Withdrawal of Phenprocoumon Therapy

**DOI:** 10.4021/cr124w

**Published:** 2011-11-20

**Authors:** Andreas Wilke, Christian M. Wende, Michael Horst, Dietmar Steverding

**Affiliations:** a Kardiologische Praxis Papenburg, Papenburg, Germany; b Abteilung Kardiologie, Marienkrankenhaus Papenburg, Papenburg, Germany; c Abteilung Kardiochirugie, Städtische Kliniken Oldenburg, Oldenburg, Germany; d Norwich Medical School, University of East Anglia, Norwich, UK

**Keywords:** Bridging therapy, Oral anticoagulant therapy, Prosthetic heart valve thrombosis

## Abstract

Patients with prosthetic heart valves require lifelong oral anticoagulant therapy based on vitamin K antagonists. These patients may need interruption of their anticoagulant therapy if they have to undergo surgery. The clinical challenge is to identify patients who can safely undergo surgery while continuing their vitamin K antagonist treatment and those who have to take short-acting heparin as part of a bridging therapy. Here we present a case of a patient with a prosthetic mitral valve whose oral anticoagulant therapy was unnecessarily discontinued by the GP prior to an upcoming cataract surgery. As a result, the patient developed thrombosis of the prosthetic mitral valve which needed to be surgically replaced.

## Introduction

Oral anticoagulant therapy using vitamin K antagonists is a very effective treatment to prevent thromboembolic complications in many medical conditions [1]. However, managing these patients when they require temporary interruption of their anticoagulant therapy because of necessary surgery is a common clinical problem. In particular, withdrawal of phenprocoumon is associated with an increase in the risk of thromboembolic complications because of the long half-life of this drug [2]. To reduce this risk, clinicians often use bridging therapy with an anticoagulant that has a much shorter half life and is therefore more easily controlled. For example, low-molecular-weight heparin is frequently used in these cases as it has a rapid onset and offset of its action compared to vitamin K antagonists [3]. Here, we report a case where such a bridging therapy went wrong causing thromboembolic complications with the result that the patient needed reoperation.

## Case Report

A 70-year-old woman with a prosthetic mitral valve replacement (St. Jude Medical mechanical valve model 29MECJ-502; year 2002) presented herself at the Kardiologische Praxis Papenburg with dyspnoea and exhaustion. For preparation of an upcoming cataract operation, her GP discontinued her anticoagulant therapy with phenprocoumon several days ago. After three days of withdrawal of any anticoagulant medication, the GP started to treat the patient once daily with subcutaneous injections of 7500 units of unfractionated sodium heparin. The patient was under this anticoagulant therapy when she presented herself at the cardiac surgery. At that time, her INR value was 1.4.

**Figure 1:**
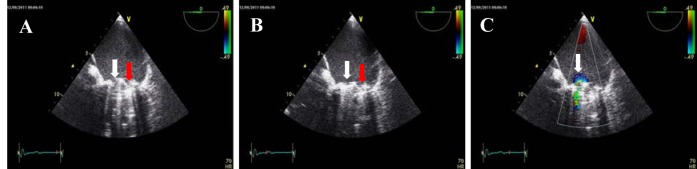
Transoesophageal echocardiography showing reduced mobility of the prosthetic valve with functional stenosis. (A) Diastole. (B) Systole. (C) Colour Doppler of the diastole. The white and red arrows point to the mobile and immobile leaflets, respectively.

**Figure 2:**
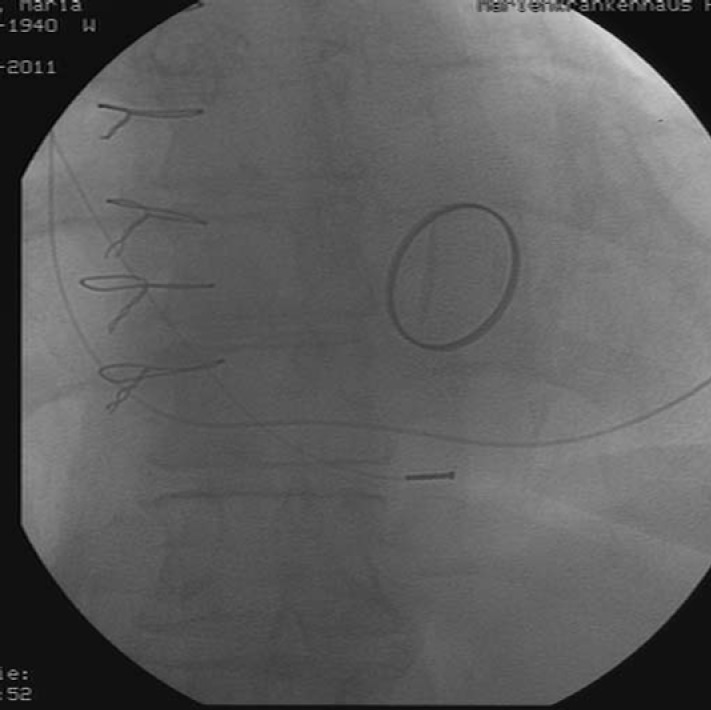
Radioscopy. Only one leaflet of the bileaflet valves opens.

**Figure 3:**
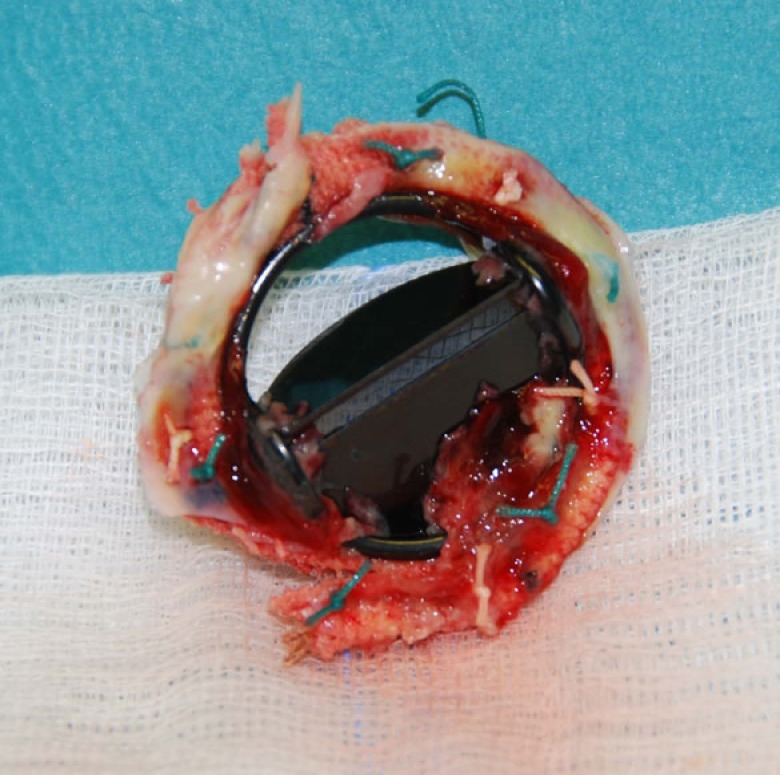
Excised valve. Thrombotic deposits are clearly visible.

Transthoratic echocardiography revealed a reduced mobility of the prosthetic mitral valve. On transoesophageal echocardiography, we confirmed the suspicion of a functional stenosed heart valve through blockage of the hinge-joint of one leaflet (Fig. 1). The patient was treated straightaway with subcutaneous injections of 8000 units of certoparin twice daily and was admitted to the hospital. Radioscopy confirmed the suspicion of a partially thrombosed mitral valve with obstruction of one leaflet of the bileaflet valve (Fig. 2). The damaged valve was surgically replaced with a bioprosthetic valve (Medtronic Mosaic Procine Bioprosthesis Model 310, diameter 31 mm). The excised valve clearly showed signs of deposits of thrombi on its rim (Fig. 3). From the atrial aspect, thrombotic deposits can be found on the hinge-joints and alongside one leaflet. Thrombotic material was also deposited on the ventricular side of the valve.

## Discussion

Surgery of patients with prosthetic heart valves demands a close monitoring of their coagulation status [4]. In the presented case, the approach of the GP was erroneous and led to the need for valve replacement in the patient [5]. Strictly speaking, the GP made two errors in the management of the patient (however, we have not spoken to the GP and therefore we do not know why the GP chose a particular option and for what reasons). First, there was no need to withdraw the anticoagulant medication for the upcoming cataract surgery in the first place [6]. The required retrobulbar anaesthesia could have been carried out under ongoing anticoagulant therapy without any problem. Alternatively, a topical anaesthesia via eye drops would have been possible. Second, even if there were good reasons to stop the anticoagulant medication, it was a poor choice to use unfractionated heparin at that dose and route of administration (subcutaneous injection of 7500 units) as bridging anticoagulation therapy. The pharmacokinetics of unfractionated heparin is unpredictable and it is very unlikely that sufficient amount of this anticoagulant agent would have been absorbed to provide effective dose from the subcutaneous injection. Instead, better controllable low-molecular-weight heparins are the anticoagulant of choice for bridging therapies [5, 7]. Before introducing any bridging therapy, e.g. the withdrawal of oral anticoagulant medicine and administration of low-molecular-weight heparins, the individual risk of thrombosis has to be considered. Medical disorders which need oral anticoagulant medication can be categorised into conditions with high (> 10%/year without anticoagulant therapy), medium (4 - 10%/year without anticoagulant therapy) and low (< 4% without anticoagulant therapy) thromboembolic risk [7]. Factors which increase the thromboembolic risk are atrial fibrillation with concomitant cardiac insufficiency, prosthetic heart valves, and recent (within the last month) arterial or venous thromboembolic events [5, 7]. The thromboembolic risk determines what course of bridging therapy is appropriate [8]. A scheme for bridging therapy with certoparin (Mono-Embolex®) as an example for anticoagulant patients with a high thromboembolic risk is shown in Table 1. Deviation from this scheme bears the risk of formation of thrombi including the consequences described in this case report. Two other examples of low-molecular-weight heparins that are suitable for bridging therapy are enoxaparin (Clexane®) and tinzaparin (Innohep®). For medical conditions with low thromboembolic risk, no bridging therapy is necessary if the anticoagulant medication is only discontinued for a short period of time [7]. Under these circumstances, a standard thrombosis prophylaxis appropriate for the particular surgery is sufficient.

**Table 1:** Example for Bridging Therapy With Certoparin 8000

Time	Vitamin K antagonist	Certoparin 8000
10 days before surgery	No	No
INR < 2.5	No	Yes, twice daily
1 day before surgery	No	Yes, once daily
Day of surgery	No	Yes, once daily
1 day after surgery and as long as there is an increased bleeding risk	No	Yes, twice daily
As soon as there is no increase bleeding risk	Yes	Yes, twice daily
INR > 3.0	Yes	No
